# Examining the Factors That Influence African Americans in the Midwest to Reduce Salt Intake

**DOI:** 10.1089/heq.2019.0079

**Published:** 2020-05-12

**Authors:** Ni Zhang, Emily Leary, Michelle Teti, Jon Stemmle, Natalie Hampton

**Affiliations:** ^1^ Department of Public Health and Recreation, San Jose State University, San Jose, California, USA.; ^2^ Department of Orthopaedic Research, University of Missouri, Columbia, Missouri, USA.; ^3^ Department of Health Sciences, University of Missouri, Columbia, Missouri, USA.; ^4^ Health Communication Research Center, University of Missouri, Columbia, Missouri, USA.

**Keywords:** salt intake, African American, cardiovascular health, hypertension

## Abstract

**Purpose:** Salt intake is associated with cardiovascular diseases that are the leading cause of death especially among African American communities in the Midwest. Interventions need to be developed to address the culture of this population to decrease the health disparities of cardiovascular disease. This study applying the Health Belief Model aims to explore the factors that are associated with the behavior of reducing salt intake among this population.

**Methods:** Three hundred ninety-nine African American adults participated in the telephone surveys. Logistic regression analysis was performed.

**Results:** We found that affective risk perception in the form of concern of salt intake as well as self-efficacy were associated with the behavior of reducing salt intake among this population. However, seeing advertisement on mass media about the effect of eating too much salt and talking to anyone about heart problems or high blood pressure issues could not influence their behavior of reducing salt intake.

**Conclusion:** This study shed light on how public health practitioners can potentially persuade African American population in Midwest to reduce salt intake through designing culturally appropriate interventions educating them about the risk of eating too much salt and increase their confidence in reducing salt in community settings.

## Introduction

### Background

Reducing salt intake is an important and cost-effective way for reducing hypertension and the risk of cardiovascular diseases.^1^ Worldwide, salt consumption has been attributed to 1.65 million annual deaths from cardiovascular causes.^2^ Studies have repeatedly shown that reductions in salt and increases in potassium consumption can improve physical health.^3^ Owing to an increase in hypertension rates as well as a related increase in the use of medications to treat hypertension,^4^ the U.S. Dietary Guidelines for Americans have recommended global reductions in salt intake.^3^

Salt is the source of the majority of dietary salt intake in Americans, and it is found in many packaged and processed foods and beverages.^5^ Most American adults consume more salt than they should.^6^ Data from the National Health and Nutrition Examination Survey (NHANES) indicate that Americans ≥2 years consume on average 3400 mg of salt each day,^7^ which is 48% higher than the recommended daily amount of 2300 mg.^8^

African American communities in general are consuming more salt than the rest of the American society.^9^ Some cultural aspects might contribute to the high salt intake in this population. African Americans' soul food, an African American cultural cuisine (e.g., butter beans [immature lima beans, usually cooked in butter], catfish [dredged in seasoned cornbread and fried], fatback [fatty cured salted pork used to season meats and vegetables]), and the general diet of African Americans tends to include large amounts of salt.^10^ Also in their culture, African Americans in general accept larger body sizes and feel less guilty about overeating than other ethnic groups.^11^

As health promotion practitioners have attempted to reduce salt intake, studies that have specifically targeted African Americans to reduce their salt intake in the United States are scarce. To prevent hypertension among this specific group of African Americans in the Midwest, we need to understand what factors affect them to reduce salt intake. This study applying Health Belief Model^12^ aims to fill the gap to examine how we can persuade African Americans in the greater Kansas City area in the Midwest to reduce salt intake.

### Health Belief Model

**FIG. 1.**
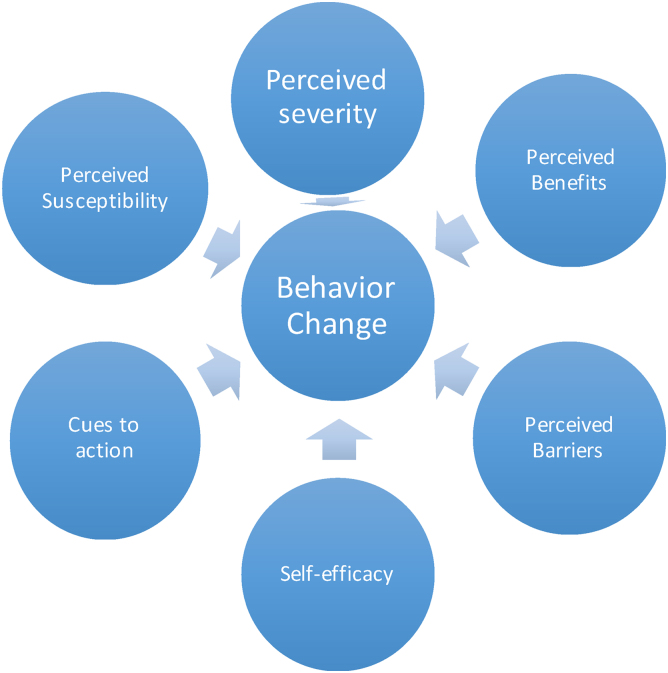
Health Belief Model.^12^

To explore the factors associated with reducing salt intake behavior among African American communities in the Midwest, this study applied the Health Belief Model.^12^ In the literature, there has been some evidence for applying the HBM in community settings,^13^ and with patients on hemodialysis who need to observe a restricted salt diet.^14^ HBM posits that people's health behavior is influenced by their perceived risks of getting the disease, the perceived severity of the disease, the perceived barriers, perceived benefits, as well as the self-efficacy of performing health behavior (Fig. 1).

HBM posits that people will change behavior when they perceive the risk of the contracting an illness or health condition, or leaving it untreated, is high.^12^ Apart from cognitive risk perception (i.e., cognitive evaluation of the hazard) that is posited in the HBM, other researchers^15^ claimed the importance of affective risk perception, which was measured by the level of worry or anxiety about the risk, in predicting behavior change.^16^

According to the HBM,^12^ perceived benefits, which are beliefs about positive features or advantages of a recommended action to reduce threat, are positively associated with performing the health behavior. In contrast, perceived barriers, which are possible obstacles associated with performing the behavior, are negatively associated with performing the health behavior. Self-efficacy, one's confidence in performing the health behavior, is also a factor as individuals who see themselves at risk for a disease or illness may want to prevent such an illness but may not *believe* he or she can take action.^12^ Cues to action that can trigger actions are important constructs since small factors in everyday life can help motivate individuals to take actions to change their behavior.^12^ These include things such as flyers, posters in a health care setting, newspaper articles, or simply knowing someone afflicted with the illness in question.

### Hypotheses

**FIG. 2.**
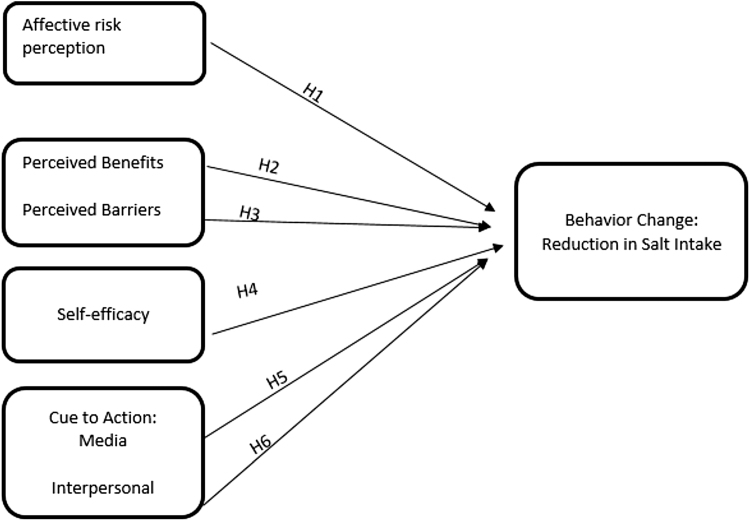
Conceptual model.

We hypothesize that African Americans who are concerned with the amount of salt in their diet (H1), who perceive more benefits (H2), less barriers to reducing salt intake (H3), and higher self-efficacy to reduce salt intake (H4) are more likely to take actions to reduce the salt intake. In addition, we hypothesize that cues to action both in the form of mass communication through advertisement (H5), and in the form of interpersonal communication (H6) are positively associated with taking actions to reduce salt. The conceptual model for this study is illustrated in Figure 2.

## Methods

### Data collection

This telephone survey was collected from January 31 through April 14, 2013. The target population were people living in or near seven zip codes in the greater Kansas City area (64109, 64110, 64127, 64128, 64129, 64130, and 64131). We prioritized this geographic population because areas of the greater Kansas City region were identified by the local health department and community partners where deaths were higher due to hypertension.^16^

The sampling design followed a two-stage probability sampling plan: the targeted zip codes in the Kansas City area were first grouped as primary sampling units, then a random sample of telephone numbers proportional to the population size was generated for each of the zip codes as secondary sampling units. Both landline and cell phone numbers were included in the sample. To increase participation, snowball sampling was later added to the original sampling design.

For landline phone numbers, the Troldahl–Carter–Bryant (TCB) respondent selection method was used to randomly select an eligible respondent if there were more than one adult ≥18 years in a household.^17^ The TCB method ensures a proper balance of gender and ages within a household and the likelihood of within-sampling-unit noncoverage error is minimized.

Eligibility for the survey was based on residence within the six targeted zip codes and only included participants ≥18 years. Responses were kept anonymous and consent was obtained using an oral consent form. Three hundred ninety-nine African American participants were surveyed; the majority (*n*=321, 80.45%) were reached on a landline, whereas others had a cell phone available to them (*n*=18, 4.51%) or cell phone only (*n*=60, 15.04%). For their participation, respondents received a $10 gift card to Walgreens. The Institutional Review Board of the University of Missouri and the Missouri Department of Health and Senior Services (MODHSS) reviewed and approved the instruments and methods used in this study.

### Instruments

Survey questions were adapted from other validated surveys, for example, survey developed by Australian Division of World Action on Salt and Health^18^ and were further tested and reviewed by African American community leaders. The MODHSS, the Missouri Sodium Knowledge in Practice (SKIP) Partnership team, and evaluators at the Missouri School of Journalism also helped develop the survey.

#### Outcomes of interest

The outcome of interest is the presence of behaviors to reduce salt intake as measured by one question: “Please tell me if you have ever reduced salt or sodium in your diet to lower your blood pressure or keep it from going higher.” The answers were recorded as dichotomous, “Yes” or “No.”

#### Explanatory measures

Affective risk perception, perceived barriers, perceived benefits, self-efficacy, cues to action through advertisements, and cues to action through interpersonal communication were explanatory measures used in analysis. Affective risk perception was measured by one question, “I am not concerned with the amount of salt in my diet” using a 4-point Likert scale. The responses were later dichotomized into “high perceived risk,” which combines the strongly disagree and disagree choices in the original measurement, and “low perceived risk,” which combines the strongly agree and agree choices in the original measurement.

Another question, “Because fast food is pretty cheap, I probably eat more of it than I should” was used to describe perceived barriers. Perceived benefits of reducing salt intake were also measured using one question, “My health would improve if I lowered the amount of salt in my diet.” Responses for perceived barriers and perceived benefits were coded as “high perceived barriers/benefits” combining the responses strongly agree and agree from the original measurement or “low perceived barriers/benefits” combining the responses of strongly disagree and disagree in the original scale.

Self-efficacy was measured using the question, “I think that if I put my mind to it, I could reduce the amount of salt in my diet” using a 4-point Likert scale, ranging from strongly disagree to strongly agree. The responses were later dichotomized into “high self-efficacy,” which combined the strongly agree and agree responses in the original measurement, and “low self-efficacy,” which combined the strongly disagree and disagree responses in the original measurement.

Cue to action for media was measured using the question, “In the last month or so, have you seen or heard any ads about the effects of eating too much salty food?” Cue to action for interpersonal communication was measured using the question, “In the last month or so, have you talked to anyone about heart problems or high blood pressure issues?” Responses for cues to action were coded as “Yes” or “No.”

#### Covariates

Covariates included in the analysis were age, gender, income, hypertensive status, and previous knowledge about salt. Age was determined by the survey question, “How old were you on your last birthday?” Gender was recorded based on the interviewer's assessment of the participants' gender, which included a choice for “I cannot tell.” Income was determined by the survey question, “Now, I am going to read you a list of income categories. Please stop me when I reach the one that best estimates your total household income before taxes for the past year.” Responses were categorized as “less than $10,000,” “at least $10,000 but under $25, 000,” “at least $25,000 but under $50,000,” and “$50,000 or more.” Hypertensive status was determined using responses to the question, “If you are comfortable sharing, please tell me what health conditions you are currently dealing with.” If that the participant was dealing with high blood pressure (HBP) and/or heart disease as given in response to this question, then those participants were considered hypertensive. Hypertensive status was modeled as dichotomous to indicate the presence or absence of HBP and/or heart disease. Knowledge about salt content in different kinds of food was determined by adding the number of correct responses to five questions. The participant was directed to indicate if they thought bread, bacon, an apple, ketchup, and canned ravioli had “high,” “medium,” or “low” salt content.

### Analysis

The survey participants were mostly female (*n*=253, 63%) and most had <$25K per year of annual income, (*n*=243, 61%). The average age of the participants was 56.9 years and ranged from 18 to 95 years. Most participants indicated high knowledge about salt and its relationship to HBP (*n*=387, 97%), and the majority were not considered hypertensive (*n*=244, 61%; Table 1).

**Table 1.** Measures of Central Tendency or Frequency of Variables Included in Analysis (*n*=399)

Variable	*N*	%
Gender		
Male	146	36.59
Female	253	63.41
Income		
<$10K	112	28.07
$10K–$25K	131	32.83
$25K–$50K	91	22.81
$50K+	65	16.29
Hypertensive		
Yes	155	38.85
No	244	61.15
Knowledge (eating too much salt can cause high blood pressure)		
High	387	97.0
Low	12	3.0
Affective risk perception		
High	313	78.45
Low	86	21.55
Perceived benefits		
High	360	90.23
Low	39	9.77
Perceived barriers		
High	268	67.17
Low	131	32.83
Self-efficacy		
High	375	93.98
Low	24	6.02
Cue to action—(ads about the effects of eating too much salty food)		
Yes	197	49.37
No	202	50.63
Cue to action—(talk to anyone about heart problems or high blood pressure issues)		
Yes	226	56.64
No	173	43.36
Behaviors present to reduce salt intake		
Yes	339	84.96
No	60	15.04
Contact type		
Cell phone	78	19.55
Landline	321	80.45
Sampling type		
Random sampling	398	99.75
Snowball recruitment	1	0.25

Logistic regression analysis was used to predict the self-described presence or absence of at least one behavior to reduce salt intake. Covariates included in all models were gender, age, income level, hypertensive status, and knowledge regarding salt intake and health. Survey weights based upon the stratified sampling method were incorporated into the model using the maximum likelihood method of estimation. Variables included in the final logistic regression model were based on the HBM and included perceived barriers, self-efficacy, perceived benefits, perceived susceptibility, and cue to action(s). Model selection was chosen based upon the Akaike information criterion, where lower values indicate a better model fit, but was also further scrutinized based upon the likely large variable to data point ratio. All assumptions for weighted logistic regression were checked and met and nonresponse issues were investigated. Adjustments for nonresponse resulted in lower variance estimates when assuming nonuniform nonresponse (i.e., nonresponse because of the survey itself) compared with uniform nonresponse (i.e., nonresponse), but no differences in model conclusions were obtained. All analyses were completed using SAS v9.4 and logistic regression was performed using the SURVEYLOGISTIC procedure.

## Results

Using the final logistic regression model, we found that affective risk perception (H1) and self-efficacy (H4) were significantly associated with the behavior of reducing salt intake. While perceived benefits (H2), perceived barriers (H3) and cues to action, both in the form of mass communication through advertisement (H5), and in the form of interpersonal communication (H6) were not significantly associated with taking to action to reduce the salt intake. Specifically controlling for age, gender, income, hypertensive status, and knowledge about the amount of salt in foods, the presence of self-efficacy (*p*=0.0097), and affective risk perception (*p*=0.0045) were statistically significant predictors of self-described behaviors to reduce salt intake (Table 2). The largest odds ratio was observed with the self-efficacy measure. Compared with those without self-described self-efficacy, those with self-efficacy have a 3.672 increase in the odds of adaptive behaviors that reduce salt intake, all else held constant (95% confidence level=1.370–9.842). In contrast, perceived barriers, perceived benefits, cues to action of seeing advertisement about the effects of eating too much salty food, and talking to other people about hypertension and heart disease were not significantly associated with self-described behaviors to reduce salt intake (Table 2).

**Table 2.** Odds Ratio Estimates and 95% Wald Confidence Limits for Each Variable

Variable	Est.	95% CL		OR	95% CL	
		UL	LL		UL	LL
Age	0.00976	−0.0111	0.0306	1.010	0.989	1.031
Female vs. male	0.2963	−0.0629	0.6555	1.809	0.882	3.710
Income						
$10K–$25K vs. <$10K	0.0745	−0.5599	0.7089	1.338	0.503	3.560
$25K–$50K vs. <$10K	−0.0938	−0.7222	0.5346	1.131	0.418	3.062
>$50K vs. <$10K	0.2361	−0.4534	0.9256	1.573	0.540	4.584
Knowledge about relationship between salt and hypertension (high vs. low)	0.0774	−0.7575	0.9122	1.167	0.220	6.200
Affective risk perception (high vs. low)	0.5469	0.1699	0.9240	2.986	1.405	6.347
Perceived barriers (high vs. low)	−0.1132	−0.5212	0.2949	0.797	0.353	1.803
Perceived benefit (high vs. low)	0.1886	−0.2734	0.6505	1.458	0.579	3.673
Self-efficacy (high vs. low)	0.6504	0.1575	1.1433	3.672	1.370	9.842
Cue to action						
Media (yes vs. no)	0.3227	−0.0283	0.6738	1.907	0.945	3.848
Interpersonal communications (yes vs. no)	0.3540	−0.0323	0.7402	2.030	0.938	4.395

All categorical variables have reported estimates for the first category listed.

CL, confidence level; LL, lower limit; UL, upper limit.

## Discussion

This study applied HBM in understanding the predictors of reducing salt intake among a high-risk underserved African American community in Kansas City. Results from this study indicated that African Americans who are more concerned with the amount of salt in their diet were associated with self-described behaviors to reduce salt intake. The relationship between affective risk perception and reducing salt intake is consistent with the findings of Sheeran, Harris, and Epton in their meta-analysis of experimental studies that affective risk perception (also known as “anticipatory emotion”) affected several measured outcomes.^19^ Our study indicates that these effects also hold for this high-risk population. Public health practitioners should address the emotional/affective appeal when designing intervention messages to tailor toward African Americans in the Midwest. More qualitative exploratory research to examine how to make African Americans concerned about their salt intake in their culture is encouraged.

High self-efficacy was the strongest predictor of self-described behaviors to reduce salt intake among this African American sample. These results indicate that if participants feel confident that they could reduce the amount of salt they ingest, they may be better able to accomplish self-described behaviors to reduce salt intake. This result is consistent with the more general literature, in particular, the study that identified a significant relationship between diet-related self-efficacy and a healthful diet.^20^ Thus, public health practitioners should help to increase African American population's self-efficacy in reducing salt intake.

In contrast to previous studies, perceived barriers of cheap fast food and benefits of improving health were not significant predictors of behaviors to reduce salt intake in this study. The lack of significance of perceived benefits and barriers could be explained by the relatively low income level in this population, with >60% of the sample reporting an annual household income of ≤$25,000. Thus, even if participants were aware of the benefits of improving health and barriers of cheap fast food to reducing salt intake, they may not be able to do anything to stop eating cheap fast food due to low income level, so could not reduce their salt intake.

In this study, exposure to cues to action, interpersonal communication about hypertension and heart disease, and exposure to advertisement through mass media about the effects of eating too much salty food were not significant predictors of taking action to reduce salt intake. These results implied that other communication strategies need to be explored to reduce salt intake among the African American population, such as healthy eating cooking events at community gatherings.

### Health equity implications

This is one of the first studies to explore what factors can influence African Americans' salt intake to address the health disparity of high rate of hypertension and cardiovascular disease among this population. We found that public health practitioners need to increase their concern for eating too much salt. Public health practitioners should address the culture of this population that they feel less guilty about overeating,^1^ which might cause too much salt intake.

The exposure to public health messages on mass media about the effects of eating too much salt were low for this population. This may be due to the fact that these messages might not be directly tailored toward this population's culture and habit with respect to mass media.

Furthermore, talking to others about heart problems or HBP issues (interpersonal communication) was not associated with self-described behaviors to reduce salt intake in this population. This is probably because this population may not realize salt intake could be one of the contributors to heart disease or they may not know they can control salt intake to decrease the risks of heart problems or hypertension. Consistently, we found that those who had high self-efficacy of reducing salt intake were more likely to do so.

Thus, future interventions need to educate this population to increase their concern about salt intake and at the same time increase their self-efficacy to do so. For example, as this population values the sense of community and their soul food is high in salt, public health practitioners could work with the community to develop education programs about the detrimental effects of eating too much salt and share healthy cooking recipes or replacements for certain ingredients for soul food at gatherings.

We found that even though some African Americans realized that cheap fast food is a barrier to reduce salt intake, they still did not take actions to reduce the salt intake. This might be due to the low access to healthy food resulting from low income level. This could shed some light for the policy makers to provide more affordable healthy food access to this population.

#### Limitations

One major limitation of the study is the potential selection bias due to the use of landline phones. Although the selection of participants was randomized using the random digital dialing technique, we could not reach the population who do not use landline phones or cell phones. Future research could adopt other data collection methods (e.g., door-to-door survey in the community) or collaboration with local community organizations to reach a more representative sample of this population.

Owing to the limited space in the questionnaire, some measurement only consisted of one single question. However, this study involved community members in the questionnaire development. These measurements were designed based on feedback from community leaders and members to improve readability. Future research to validate these instruments are needed.

## Conclusion

This study applied the Health Belief Model to explore the factors associated with reducing salt intake among African Americans in the Midwest. Results indicate that African Americans who are concerned with their salt intake and felt sufficiently confident to control the amount of salt they take were more likely to take actions to reduce salt intake. These findings inform public health practitioners to target this high-risk underserved urban population to increase their affective risk perception as well as self-efficacy simultaneously to encourage them to reduce salt intake to reduce hypertension and cardiovascular diseases in the long run.

## Abbreviations Used

HBP: high blood pressure
MODHSS: Missouri Department of Health and Senior Services
NHANES: National Health and Nutrition Examination Survey
PSUs: primary sampling units
SKIP: Sodium Knowledge in Practice
SSUs: secondary sampling units
TCB: Troldahl–Carter–Bryant
